# Early kinetics of C-reactive protein as prognosticator for survival in a real-world cohort of patients with metastatic renal cell cancer under first-line therapy with immune checkpoint inhibitors

**DOI:** 10.1007/s12094-023-03317-z

**Published:** 2023-09-11

**Authors:** Vayda Schüttke, Cathrin Kusiek, Susanne Fuessel, Christian Thomas, Bjoern Thorben Buerk, Kati Erdmann

**Affiliations:** 1 Department of Urology, Technische Universität Dresden, Faculty of Medicine and University Hospital Carl Gustav Carus, Dresden, Germany; 2 German Cancer Consortium (DKTK), Partner Site Dresden, Dresden and German Cancer Research Center (DKFZ), Heidelberg, Germany

**Keywords:** Biomarker, CRP kinetics, Immunotherapy, Metastatic renal cell carcinoma, Prognosis, Tyrosine kinase inhibitors

## Abstract

**Purpose:**

This study investigated the prognostic potential of baseline C-reactive protein (CRP) levels and early CRP kinetics in a real-world cohort of patients with metastatic renal cell carcinoma (mRCC) under first-line (1L) therapy with immune checkpoint inhibitors (CPI).

**Methods/patients:**

Analyses were performed retrospectively in a cohort of 61 mRCC patients under CPI-based 1L therapy. Patients were stratified based on baseline CRP (< 10 vs ≥ 10 mg/l) and CRP change within the initial three months of CPI therapy (normal: baseline < 10 mg/l, normalized: baseline ≥ 10 mg/l and nadir < 10 mg/l, non-normalized: baseline and nadir ≥ 10 mg/l). Finally, the association of baseline CRP and CRP change with progression-free (PFS) and overall survival (OS) was evaluated.

**Results:**

Baseline CRP was not significantly associated with both PFS (p = 0.666) and OS (p = 0.143). Following stratification according to early CRP kinetics, 23, 25 and 13 patients exhibited normal, normalized and non-normalized CRP levels, respectively. Patients with normal and normalized CRP had a markedly prolonged PFS (p = 0.091) and OS (p = 0.008) compared to patients with non-normalized CRP. Consequently, significantly better PFS (p = 0.031) and OS (p = 0.002) were observed for the combined normal-normalized group. In multivariate analysis including ECOG and IMDC risk, normalized CRP kinetics alone or in combination with the normal group was identified as significant independent risk factor for OS, whereas a statistical trend was observed for PFS.

**Conclusions:**

The present study emphasizes the prognostic potential of early CRP kinetics in CPI-treated mRCC. As a standard laboratory parameter, CRP can be easily implemented into clinical routine to facilitate therapy monitoring.

**Supplementary Information:**

The online version contains supplementary material available at 10.1007/s12094-023-03317-z.

## Introduction

In comparison to standard anti-angiogenic therapy with tyrosine kinase inhibitors (TKI), immune checkpoint inhibitors (CPI) have improved the prognosis of patients with metastatic renal cell carcinoma (mRCC) [1, 2]. In addition to the combination of two CPI (ipilimumab/nivolumab), standard first-line (1L) therapy of mRCC patients currently comprises combinations of CPI (avelumab, nivolumab, pembrolizumab) with TKI (axitinib, cabozantinib, lenvatinib) [1, 3]. Monotherapy with the CPI nivolumab is used in subsequent treatment lines after TKI therapy [3].

Nonetheless, the response to CPI-based therapy is highly variable due to primary or secondary resistances [4]. Consequently, the identification of easy to implement prognostic and/or predictive biomarkers is highly needed in order to optimize patient benefit, minimize risk of toxicities and guide therapeutic approaches. In contrast to other cancers such as melanoma, lung and bladder cancer, tumor tissue levels of *programmed cell death ligand 1* (PD-L1) or the tumor mutational burden are insufficient to predict response to CPI treatment in mRCC [5–7]. These tissue markers would also be unsuitable for therapy monitoring as the repeated collection of tissue samples via biopsy would represent an additional burden for patients undergoing palliative therapy. Therefore, standard laboratory parameters such as C-reactive protein (CRP), which can be determined quickly and easily during the course of therapy, have come into focus for therapy monitoring of CPI-treated patients.

CRP is a circulating acute-phase protein, which is routinely determined as a representative marker for both acute and chronic systemic inflammation. Chronic inflammation can promote an immune-suppressive environment and thus, influences tumor growth, prognosis and therapy response [8, 9]. Concordantly, higher pre-operative serum CRP levels were accompanied with the infiltration of immune-suppressive cells in tissue samples from RCC patients [10]. Higher baseline CRP levels were also associated with shorter survival and worse CPI efficacy in mRCC patients under CPI-based 1L [11] or later-line therapy [12, 13].

Furthermore, the dynamic change of CRP levels (CRP kinetics) in the early therapy phase can act as an even better prognostic and predictive marker for CPI therapy in mRCC patients [14–20]. Patients are stratified by mostly two classification methods: (i) into normal, normalized and non-normalized groups according to the change of the absolute CRP value [15, 17, 18] or (ii) into responders, flare-responders and non-responders based on the relative change compared to baseline CRP [14, 16]. To date, three studies have demonstrated the prognostic and/or predictive potential of early CRP kinetics in mRCC patients under CPI-based 1L therapy [16–18]. In two real-world cohorts and one phase III trial, mRCC patients treated with ipilimumab/nivolumab, avelumab/axitinib or pembrolizumab/axitinib with normalized CRP and CRP flare-response had a significantly prolonged survival compared to patients with non-normalized CRP [17, 18] or non-responders [16], respectively. Mechanistically, normal and normalized CRP levels following systemic CPI therapy might be a sign for improved T cell response or diminished inflammation due to decreased tumor mass. Therefore, early CRP kinetics may be suited as surrogate marker for therapy efficacy.

However, the aforementioned studies investigated the prognostic potential of early CRP kinetics in mRCC 1L cohorts consisting of patients treated with only one or two different CPI-based combination therapies. In order to better reflect the current diverse treatment landscape in mRCC, studies using broader real-world cohorts including all available CPI-based 1L therapy options are needed. Therefore, this study investigated the prognostic potential of baseline CRP and early CRP kinetics in mRCC patients under CPI-based 1L therapy by using a real-world cohort implementing all therapeutic options. The two established classification methods were used to categorize patients according to early CRP kinetics and were then compared regarding their association with survival.

## Patients and methods

### Patients

Between March 2019 and October 2022, a total of 65 consecutive mRCC patients were initiated on CPI-based 1L therapy at the Department of Urology (University Hospital Dresden). Treatment selection for each patient was carried out according to the effective guidelines at the time and based on the patient’s performance status and International mRCC Database Consortium (IMDC) risk. After exclusion of four patients with missing clinical or CRP data, 61 patients were evaluated in this retrospective observational study. The respective treatment was administered according to the current guidelines until radiological or clinical disease progression, unacceptable toxicity or death from any cause. All clinical and laboratory data were obtained from the electronic database and patient medical records with a censor date of 28 February 2023. Data collection and analysis were approved by the institutional review board of the Technische Universität Dresden (BO-EK-107032023) and conducted according to the Declaration of Helsinki.

### Assessment of serum CRP and definition of early CRP kinetics

Serum CRP levels as well as parameters for kidney and liver function were measured in an accredited routine laboratory at baseline before initiation of 1L therapy (closest to 1L start, maximum 3 days before) and thereafter regularly at administration of CPI. Patients were then stratified based on the baseline CRP levels (< 10 vs ≥ 10 mg/l) and the early CRP change within the initial three months of 1L therapy (normal, normalized, non-normalized). The normal group consisted of patients with baseline CRP levels < 10 mg/l. The normalized group included patients whose baseline CRP levels were ≥ 10 mg/l and nadir CRP levels declined < 10 mg/l within the initial three months. Patients with baseline and nadir CRP levels within the initial three months ≥ 10 mg/l constituted the non-normalized group. For patients with a 1L duration < 3 months, the nadir CRP level was determined during their entire 1L treatment duration.

In addition, patients were grouped according to the classification of early CRP dynamics by Fukuda et al. into CRP responders, flare-responders and non-responders [14]. CRP flare-responders showed a more than doubling in CRP levels from baseline within the 1st month after 1L initiation (flare) and then a subsequent decrease below the baseline level within the initial three months of 1L therapy. The CRP responder group consisted of patients with a CRP decrease of ≥ 30% from baseline within three months without flare. All other patients were classified as CRP non-responders. This second classification was annotated as “CRP flare-response” to avoid confusion with the first stratification method.

### Statistical analysis

Statistical analyses were carried out with IBM SPSS Statistics 28.0.1.0 (IBM, Armonk, NY, USA) and GraphPad Prism 9.5.0 (GraphPad Software, San Diego, CA, USA). Kruskal-Wallis or Chi-square tests were used for inter-group comparisons of continuous and categorized variables, respectively. Progression-free survival (PFS) was defined as the period from the initiation of 1L therapy to disease progression or death from any cause. Overall survival (OS) was defined as the period from the start of 1L therapy until death from any cause. Patients without disease progression (PFS) or lost to follow-up (OS) were censored at the time of last confirmed survival. PFS and OS were determined by the Kaplan-Meier method and differences between groups were assessed using the log-rank test. Survival rates were calculated from life tables. Univariate and multivariate Cox regression analyses were performed to identify prognostic factors for PFS and OS. A p value < 0.05 was considered statistically significant, whereas a statistical trend was indicated by p values ≥ 0.05 and < 0.1.

## Results

### Patient cohort

Overall, 61 patients with mRCC receiving either CPI + TKI (n = 45, 73.8%) or CPI + CPI (ipilimumab/nivolumab; n = 16, 26.2%) as 1L therapy were included in this study (Table 1). The CPI + TKI group included the following drug combinations: avelumab/axitinib (n = 17), nivolumab/cabozantinib (n = 2), pembrolizumab/axitinib (n = 16) and pembrolizumab/lenvatinib (n = 10). Patients were mostly male (70.5%) and had a median age of 65 years, an Eastern Cooperative Oncology Group performance status (ECOG) of ≤ 1 (80.4%) and an intermediate or poor IMDC risk (86.9%). The median time from histological diagnosis of (m)RCC to initiation of 1L therapy was 4.6 months with the majority of the patients having been diagnosed with clear cell RCC (91.8%), of whom six patients also showed a sarcomatoid differentiation. At the start of 1L therapy, most patients presented with multiple metastasis (75.4%) with lung, lymph nodes and bone as most common metastatic sites. Most patients also had a partial or radical nephrectomy (63.9%) at a median 17.5 months prior to 1L.

**Table 1:** Patient characteristics of the total cohort and sub-cohorts according to early CRP kinetics

Parameter	Category	Total cohort	Early CRP kinetics			P value^b^
			Normal	Normalized	Non-normalized	
Patients (n)		61 (100.0%)	23 (37.7%)	25 (41.0%)	13 (21.3%)	
1L therapy (n)	CPI + TKI	45 (73.8%)	17 (27.9%)	21 (34.4%)	7 (11.5%)	0.134^c^
	CPI + CPI	16 (26.2%)	6 (9.8%)	4 (6.6%)	6 (9.8%)	
Age (years)	Median (range)	65 (47–82)	65 (52–76)	64 (47–82)	67 (56–75)	0.853^d^
Sex (n)	Male	43 (70.5%)	18 (29.5%)	19 (31.2%)	6 (9.8%)	**0.094** ^c^
	Female	18 (29.5%)	5 (8.2%)	6 (9.8%)	7 (11.5%)	
ECOG (n)	0	22 (36.1%)	10 (16.4%)	10 (16.4%)	2 (3.3%)	0.188^c^
	1	27 (44.3%)	11 (18.0%)	8 (13.1%)	8 (13.1%)	
	≥ 2	12 (19.7%)	2 (3.3%)	7 (11.5%)	3 (4.9%)	
IMDC risk (n)	Favorable	8 (13.1%)	6 (9.8%)	2 (3.3%)	0 (0.0%)	**0.005**^c^
	Intermediate	29 (47.5%)	13 (21.3%)	13 (21.3%)	3 (4.9%)	
	Poor	24 (39.3%)	4 (6.6%)	10 (16.4%)	10 (16.4%)	
Time from 1st diagnosis to 1L (months)	Median (range)	4.6 (0.2–295.5)	11.0 (0.5–101.9)	2.1 (0.2–139.4)	3.6 (0.4–295.5)	0.322^d^
Clear cell histology (n)	Yes	56 (91.8%)	22 (36.1%)	22 (36.1%)	12 (19.7%)	0.626^c^
	No/unknown	5 (8.2%)	1 (1.6%)	3 (4.9%)	1 (1.6%)	
Metastatic organs at 1L (n)	Single	15 (24.6%)	9 (14.8%)	5 (8.2%)	1 (1.6%)	**0.086**^c^
	Multiple	46 (75.4%)	14 (22.9%)	20 (32.8%)	12 (19.7%)	
Prior nephrectomy (n)	No	22 (36.1%)	4 (6.6%)	11 (18.0%)	7 (11.5%)	**0.051**^c^
	Yes	39 (63.9%)	19 (31.2%)	14 (22.9%)	6 (9.8%)	
Time from prior nephrectomy to 1L (months)	Median (range)	17.5 (1.0–295.5)	17.0 (1.0–101.9)	16.7 (1.9–139.4)	49.4 (4.5–295.5)	0.373^d^
Follow-up duration (months)	Median (range)	12.4 (1.2–41.1)	15.8 (1.5–33.6)	13.3 (1.2–38.2)	8.0 (1.3–41.1)	0.505^d^
Baseline CRP (mg/l)	Median (range)	20.2 (0.7–241.9)	2.7 (0.7–8.6)	26.6 (10.3–197.2)	86.5 (21.2–241.9)	** < 0.001**^d^
Nadir CRP (mg/l)^a^	Median (range)	4.8 (0.6–133.9)	2.5 (0.6–9.2)	4.2 (1.0–9.8)	29.6 (12.1–133.9)	** < 0.001**^d^
CRP measurements (n)^a^	Median (range)	7 (2–11)	7 (3–11)	7 (2–11)	7 (3–10)	0.973 ^d^

^a^Within first three months after initiation of 1L therapy

^b^Comparison between groups of early CRP kinetics by ^c^Chi-square or ^d^Kruskal-Wallis test

Significant p values (< 0.05) and statistical trends (p ≥ 0.05 and < 0.1) are displayed in bold

The median follow-up time, PFS and OS after initiation of 1L therapy were 12.4, 16.1 and 35.6 months, respectively. Thirty (49.2%) patients remained under 1L therapy, whereas 21 (34.4%) and 10 (16.4%) patients had progressed or died under 1L therapy, respectively. Overall, 19 patients (31.1%) had died until the end of data collection (February 2023).

### Classification of patients according to baseline CRP levels and early CRP kinetics

Although the median CRP level at baseline was 20.2 mg/l (Table 1), patients were categorized into groups with low and high baseline CRP levels using 10 mg/l as cutoff value, which is similar to other studies [11, 12]. Subsequently, 23 patients (37.7%) had a low and 38 patients (62.3%) had a high baseline CRP level.

Following stratification according to early CRP kinetics [15, 17, 18], 23 (37.7%), 25 (41.0%) and 13 (21.3%) patients exhibited normal, normalized and non-normalized CRP levels, respectively. As expected, patients of the normal group showed significantly lower baseline CRP levels than patients of the normalized and non-normalized group (Table 1). In addition, the CRP nadir after initiation of 1L therapy was significantly lower in the normal and normalized CRP groups compared to the non-normalized group (Table 1). The median time from 1L initiation until CRP normalization was 4.7 weeks (range 1.9–12.0 weeks). Of note, the median number of CRP measurements within the first three months after initiation of 1L therapy was equal (n = 7) in all three groups. No significant differences were detected regarding the baseline and nadir CRP levels as well as the number of CRP measurements depending on the time point of initiation of 1L therapy (Table S1).

Except for IMDC risk, no significant differences between the three CRP groups could be observed for the demographic and clinico-pathological parameters as well as for the follow-up duration of the patients (Table 1). Interestingly, patients of the normal CRP group had worse kidney function at baseline as reflected by a higher creatinine level and lower filtration rate (Table S2), which might be attributable to the per trend higher frequency of prior nephrectomies in these patients (p = 0.051; Table 1). Furthermore, laboratory parameters for liver function did not differ between groups of early CRP kinetics (Table S2).

In addition, patients were grouped according to the CRP dynamics classification by Fukuda et al. [14] resulting in 30 CRP responders (52.6%), 13 flare-responders (22.8%) and 14 non-responders (24.6%). Four patients could not be categorized due to missing CRP data at critical time points needed for this classification. The median time from 1L initiation until response was 3.0 weeks (range 0.6–10.3 weeks). The CRP flare occurred at a median time of 1.9 weeks (range 0.1–4.0 weeks) with a subsequent decrease below baseline CRP at 6.0 weeks (range 2.3–12.1 weeks) after start of 1L.

### Association of baseline CRP levels and early CRP kinetics with survival

**Fig. 1:**
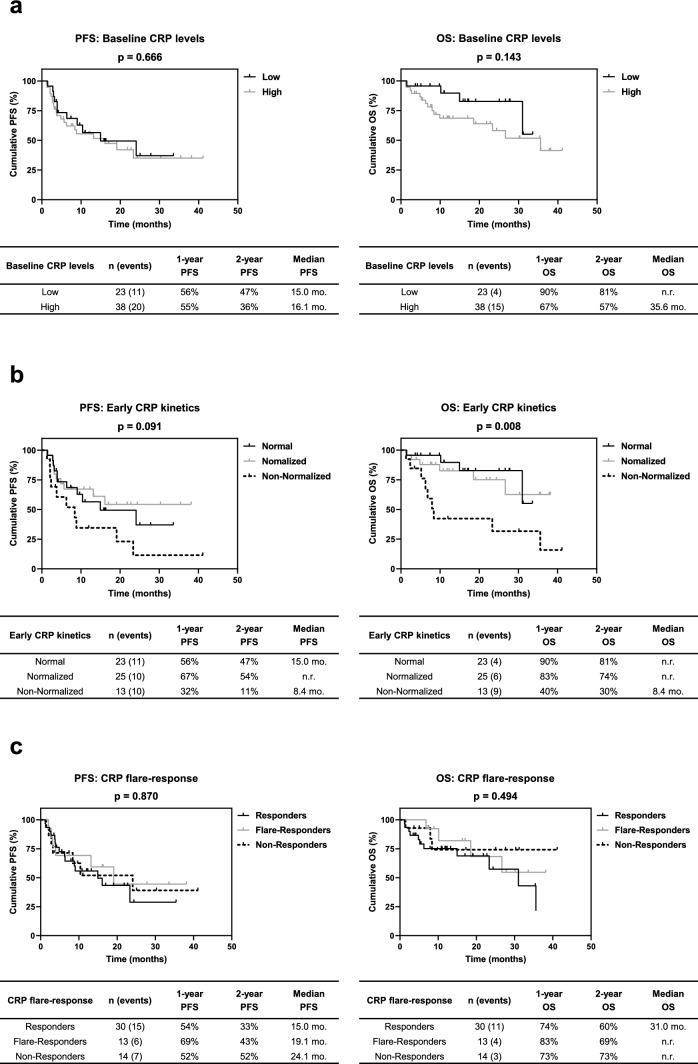
Association of **a** baseline CRP levels, **b** early CRP kinetics and **c** CRP flare-response classification with PFS and OS after initiation of CPI-based 1L therapy of mRCC patients. The table beneath each Kaplan-Meier curve includes the number of patients and events in each category as well as the respective median survival times, 1- and 2-year survival rates. P values were calculated by the log-rank test. Abbreviations:* mo.* months; *n.r.* not reached

Baseline CRP levels were not significantly associated with PFS (p = 0.666) and OS (p = 0.143), although patients with low baseline CRP levels showed a markedly prolonged OS compared to patients with high baseline CRP levels (Fig. 1a).

**Fig. 2:**
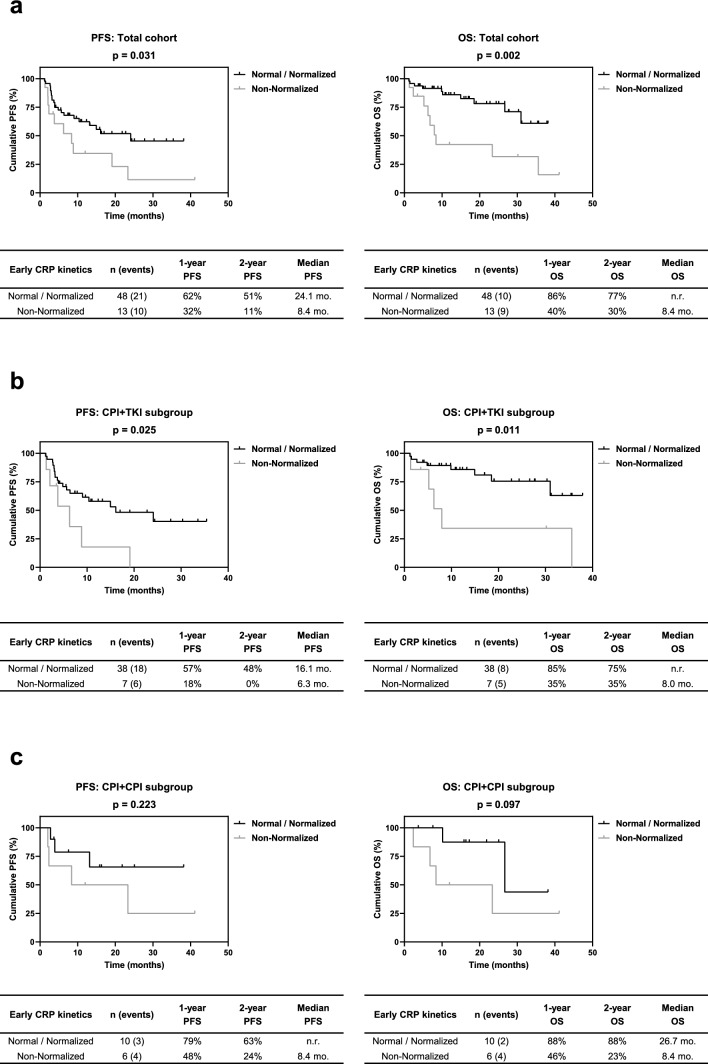
Association of early CRP kinetics (two groups: normal/normalized vs non-normalized) in **a** the total cohort as well as in the treatment subgroups of **b** CPI + TKI and **c** CPI + CPI with PFS and OS after initiation of CPI-based 1L therapy of mRCC patients. The table beneath each Kaplan-Meier curve includes the number of patients and events in each category as well as the respective median survival times, 1- and 2-year survival rates. P values were calculated by the log-rank test. Abbreviations: *mo.* months; *n.r.* not reached

Patients with normal and normalized CRP showed per trend a longer PFS (p = 0.091) and a significantly longer OS (p = 0.008) than patients with non-normalized CRP (Fig. 1b). Significantly prolonged PFS and OS could be observed after combining the normal and normalized CRP groups (p = 0.031 & p = 0.002; Fig. 2a). These associations were reflected by higher 1-year and 2-year survival rates. In the CPI + TKI subgroup, patients with normal or normalized CRP alone or in combination also exhibited a markedly longer PFS (p = 0.078 & p = 0.025) and OS (p = 0.038 & p = 0.011) than patients with non-normalized CRP (Figs. S1a and 2b). A similar trend was observed in the CPI + CPI subgroup, but without reaching statistical significance due to lower patient numbers (Figs. S1b & 2c). The analysis of further subgroups of the CPI + TKI group was forgone due to the low number of patients in these subgroups (n = 2–17), which partly resulted in CRP kinetics groups without patient assignments.

Interestingly, categorizing patients into CRP responders, flare-responders and non-responders was not associated with PFS (p = 0.870) and OS (p = 0.494) (Fig. 1c).

### Cox regression analysis for baseline CRP levels and early CRP kinetics

Univariate Cox regression analysis confirmed the results of the survival analysis for baseline CRP and CRP dynamics for PFS and OS (Tables 2 and 3). Neither baseline CRP nor CRP flare-response emerged as significant factors for PFS and OS (p > 0.1). In contrast, patients with normalized CRP had a reduced risk for progression of about 60% (p = 0.044) compared to patients with non-normalized CRP (Table 2). Furthermore, patients with normal and normalized CRP had a significantly diminished risk for death of about 80% (p = 0.014) and 70% (p = 0.021), respectively (Table 3). Of the evaluated clinico-pathological parameters, only ECOG (p = 0.082) and IMDC risk (p = 0.019) showed a statistical trend or significance in univariate Cox regression analysis for OS but not for PFS (Tables 2 and 3). Nevertheless, a basic model including ECOG and IMDC risk was used for multivariate Cox regression analysis of early CRP kinetics for both PFS and OS. Subsequently, normalized CRP kinetics remained per trend a prognostic factor for PFS (p = 0.088) and was identified as independent prognosticator for OS (p = 0.046), whereas none of the included clinico-pathological parameters reached statistical significance (Tables 2 and 3). Strikingly, similar results were obtained for both PFS and OS following combination of the normal and normalized CRP group (Table 4).

**Table 2:** Univariate and multivariate Cox regression analyses for PFS dependent on clinico-pathological parameters as well as on baseline CRP, early CRP kinetics and CRP flare-response classification

Parameter	Category	PFS univariate		PFS multivariate	
		HR (95% CI)	P value	HR (95% CI)	P value
Age	Continuous	0.993 (0.951–1.037)	0.748		
Sex	Male	Reference	0.566		
	Female	0.790 (0.353–1.768)			
ECOG	0	Reference	0.288	Reference	0.462
	1	1.675 (0.716–3.921)	0.234	1.374 (0.553–3.411)	0.493
	≥ 2	2.204 (0.797–6.090)	0.128	1.933 (0.684–5.467)	0.214
IMDC risk	Favorable/intermediate	Reference	0.422	Reference	0.948
	Poor	1.347 (0.650–2.791)		1.028 (0.449–2.354)	
Prior nephrectomy	No	Reference	0.660		
	Yes	0.847 (0.403–1.777)			
1L therapy	CPI + TKI	Reference	0.408		
	CPI + CPI	0.700 (0.301–1.629)			
Metastatic organs at 1L	Single	Reference	0.219		
	Multiple	1.774 (0.711–4.423)			
Baseline CRP	Low	Reference	0.667		
	High	1.176 (0.563–2.456)			
Early CRP kinetics	Non-normalized	Reference	0.104	Reference	0.222
	Normal	0.490 (0.208–1.157)	0.104	0.557 (0.214–1.450)	0.230
	Normalized	0.405 (0.168–0.976)	**0.044**	0.449 (0.179–1.126)	**0.088**
CRP flare-response	Non-responder	Reference	0.871		
	Responder	1.089 (0.440–2.699)	0.853		
	Flare-responder	0.844 (0.283–2.518)	0.761		

Significant p values (< 0.05) and statistical trends (p ≥ 0.05 and < 0.1) are displayed in bold

*HR* hazard ratio, *CI* confidence interval

**Table 3:** Univariate and multivariate Cox regression analyses for OS dependent on clinico-pathological parameters as well as on baseline CRP, early CRP kinetics and CRP flare-response classification

Parameter	Category	OS univariate		OS multivariate	
		HR (95% CI)	P value	HR (95% CI)	P value
Age	Continuous	1.024 (0.969–1.082)	0.396		
Sex	Male	Reference	0.794		
	Female	0.872 (0.312–2.437)			
ECOG	0	Reference	**0.082**	Reference	**0.094**
	1	1.042 (0.333–3.259)	0.943	0.540 (0.150–1.939)	0.345
	≥ 2	3.164 (0.958–10.452)	**0.059**	1.926 (0.561–6.616)	0.298
IMDC risk	Favorable/intermediate	Reference	**0.019**	Reference	0.138
	Poor	3.040 (1.204–7.672)		2.276 (0.768–6.741)	
Prior nephrectomy	No	Reference	0.247		
	Yes	0.581 (0.231–1.458)			
1L therapy	CPI + TKI	Reference	0.731		
	CPI + CPI	1.186 (0.449–3.130)			
Metastatic organs at 1L	Single	Reference	0.232		
	Multiple	2.133 (0.616–7.390)			
Baseline CRP	Low	Reference	0.154		
	High	2.247 (0.739–6.836)			
Early CRP kinetics	Non-Normalized	Reference	**0.016**	Reference	**0.095**
	Normal	0.225 (0.068–0.743)	**0.014**	0.339 (0.090–1.270)	0.108
	Normalized	0.296 (0.105–0.835)	**0.021**	0.328 (0.110–0.980)	**0.046**
CRP flare- response	Non-Responder	Reference	0.504		
	Responder	1.957 (0.544–7.039)	0.304		
	Flare-Responder	1.226 (0.274–5.484)	0.790		

Significant p values (< 0.05) and statistical trends (p ≥ 0.05 & < 0.1) are displayed in bold

*HR* hazard ratio, *CI* confidence interval

**Table 4:** Univariate and multivariate Cox regression analyses for PFS and OS dependent on selected clinico-pathological parameters and early CRP kinetics (two groups: normal / normalized vs non-normalized)

Parameter	Category	PFS univariate		PFS multivariate	
		HR (95% CI)	P value	HR (95% CI)	P value
ECOG	0	Reference	0.288	Reference	0.469
	1	1.675 (0.716–3.921)	0.234	1.399 (0.565–3.463)	0.468
	≥ 2	2.204 (0.797–6.090)	0.128	1.921 (0.679–5.430)	0.218
IMDC risk	Favorable/intermediate	Reference	0.422	Reference	0.957
	Poor	1.347 (0.650–2.791)		0.978 (0.441–2.171)	
Early CRP kinetics	Non-normalized	Reference	**0.036**	Reference	**0.092**
	Normal/normalized	0.446 (0.209–0.949)		0.493 (0.216–1.123)	

		OS univariate		OS multivariate	
		HR (95% CI)	P value	HR (95% CI)	P value
ECOG	0	Reference	**0.082**	Reference	**0.092**
	1	1.042 (0.333–3.259)	0.943	0.541 (0.152–1.931)	0.344
	≥ 2	3.164 (0.958–10.452)	**0.059**	1.926 (0.561–6.616)	0.298
IMDC risk	Favorable/intermediate	Reference	**0.019**	Reference	0.127
	Poor	3.040 (1.204–7.672)		2.260 (0.793–6.441)	
Early CRP kinetics	Non-normalized	Reference	**0.004**	Reference	**0.030**
	Normal/normalized	0.264 (0.106–0.656)		0.332 (0.123–0.898)	

Significant p values (< 0.05) and statistical trends (p ≥ 0.05 & < 0.1) are displayed in bold

*HR* hazard ratio, *CI* confidence interval

## Discussion

Based on the close connection between chronic inflammation and tumorigenesis, inflammatory markers, such as CRP, might be helpful to predict and prognosticate tumor response to CPI-based therapy [9]. In the present study, however, baseline CRP levels were not significantly associated with both PFS and OS, although patients with baseline CRP levels ≥ 10 mg/l showed a noticeably shorter OS than patients with low baseline CRP levels. In accordance, higher baseline CRP levels ≥ 10 mg/l were significantly associated with shorter OS, but not PFS in mRCC patients treated by 1L ipilimumab/nivolumab [11] or ≥ 2L nivolumab [12].

A marked association with survival could be observed when patients were stratified according to the dynamic change of CRP levels into groups of normal, normalized and non-normalized CRP. Patients with normal and normalized CRP had per trend a prolonged PFS and a significantly longer OS than patients with non-normalized CRP. This prognostic association could also be demonstrated in the treatment subgroups of CPI + TKI and CPI + CPI, albeit not always reaching statistical significance. In multivariate analysis including ECOG and IMDC risk, normalized CRP kinetics alone or in combination with the normal group was identified as significant independent risk factor for OS, whereas a statistical trend was observed for PFS. Compared to ECOG and IMDC risk, CRP kinetics proved to be a superior prognosticator for survival in the present cohort. In accordance, mRCC patients from a phase III trial treated by 1L avelumab/axitinib with normal and normalized CRP exhibited a significantly longer PFS and OS as well as better response rates than patients with non-normalized CRP [18]. Similar to our findings, early CRP kinetics were also identified as an independent prognostic factor for OS and per trend for PFS in a multivariate analysis. Furthermore, Tachibana et al. showed that mRCC patients treated by 1L ipilimumab/nivolumab with normal and normalized CRP had a significantly longer PFS and higher objective response rates than patients with non-normalized CRP [17]. However, they did not report on OS. In ≥ 2L nivolumab monotherapy, patients with normal and normalized CRP levels also exhibited a prolonged survival and improved response rates [15, 19, 20].

The stratification of CPI-treated mRCC patients based on the absolute CRP change might hold a clinical value for early therapy adjustments. Both patient groups with a prognostic advantage can be identified very early on: patients with normal CRP already at baseline and patients with normalized CRP at around 4.7 weeks on-treatment in the present cohort. In contrast, routine radiological staging is usually performed eight to 12 weeks after therapy initiation. Therefore, an early response evaluation and in turn the chance for prompt staging and therapy adjustments might be possible with early CRP kinetics.

The underlying mechanisms of CRP normalization have not been fully elucidated yet. CRP production in the liver is mostly stimulated by interleukin-6 (IL-6) [21], which is also expressed by RCC cells and acts as an autocrine growth factor in RCC [22–25]. Consequently, the reduction of tumor mass through systemic anti-tumor therapy could lead to diminished IL-6 production and subsequently to CRP normalization. Accordingly, high serum levels of IL-6 were associated with worse oncological outcome in mRCC patients treated by 1L pembrolizumab/axitinib [26]. Since it has been shown that higher CRP levels are reflective of an immune-suppressive tumor microenvironment in RCC [10], normal and normalized CRP levels in CPI-treated patients might be associated with an improved T cell response. Taken together, early CRP kinetics and particularly CRP normalization could function as surrogate marker for therapy efficacy.

In addition to the stratification according to the dynamic change of the absolute CRP value, patients were also classified into responders, flare-responders and non-responders based on the relative CRP change. This classification was not associated with PFS and OS in the present study. In other studies, however, CRP flare-responders exhibited prolonged survival and improved response rates in mRCC patients treated by ipilimumab/nivolumab or pembrolizumab/axitinib in 1L [16] or by nivolumab in ≥ 2L [14]. Similar results were observed in other tumor entities treated by CPI such as lung and bladder cancer [27–30].

In order to unravel possible discrepancies, the respective patient subgroups according to absolute and relative CRP change were cross-tabulated (Table 5). Overall, approximately 75% of the patients with normalized CRP levels were also identified as responders, whereas flare-responders were somewhat evenly distributed across the normal, normalized and non-normalized groups (~ 15–29%). However, most strikingly, almost 50% of the patients with normal CRP levels were categorized as non-responders and more than 60% of the patients with non-normalized CRP levels as responders despite continuously elevated CRP levels ≥ 10 mg/l after treatment start. Interestingly, patients with normal CRP mismatched as non-responders had a significantly longer OS than patients with non-normalized CRP mismatched as responders (Fig. S2). A similar effect could be observed for PFS, but did not reach significance (Fig. S2). These findings indicate a more robust classification of patients based on the absolute CRP change with regards to prognosis. In contrast, the CRP flare-response classification as proposed by Fukuda et al. categorizes patients relative to the baseline CRP level regardless if the baseline CRP was rather high or low [14], which in turn might lead to prognostic misclassification in some cases.

**Table 5:** Patient distribution according to early CRP kinetics (normal, normalized, non-normalized) and CRP flare-response classification (responders, flare-responders, non-responders)

	Normal^a^	Normalized^a^	Non-normalized^a^	Total^a^
Responders	5 (23.8%)	17 (73.9%)	8 (61.5%)^b^	30 (52.6%)
Flare-Responders	6 (28.6%)	5 (21.7%)	2 (15.4%)	13 (22.8%)
Non-Responders	10 (47.6%)^b^	1 (4.3%)	3 (23.1%)	14 (24.6%)
Total	21 (100%)	23 (100%)	13 (100%)	57 (100%)

^a^Percentages are depicted according to the total of each column

^b^Potentially mismatched subgroups of CRP kinetics

Although the present study further emphasizes the prognostic potential of early CRP kinetics in mRCC treated by CPI, several limitations should be noted. The data were collected retrospectively using a relatively small patient cohort and missing data points for CRP could not be prevented. Nonetheless, the baseline clinico-pathological features as well as the median follow-up time, PFS and OS of the present cohort were similar to a large real-world study including 729 mRCC patients under CPI-based 1L therapy, which retrospectively analyzed survival benefits [31]. Furthermore, it should be noted that the present real-world cohort included all available CPI-based 1L therapy options for mRCC and thus, better reflected the current diverse treatment landscape compared to larger phase III trials investigating only specific drugs or drug combinations. Higher patient numbers are required to further validate the prognostic usefulness of early CRP kinetics and to perform analysis for specific drug combinations, particularly those with recent approval (e.g., nivolumab/cabozantinib, pembrolizumab/lenvatinib). A larger prospective study under real-world conditions is needed for the verification of the best CRP cutoff value as well as of the optimal period and frequency for CRP measurements. Finally, the association with therapy outcome was not investigated in the present study, albeit the significantly prolonged survival of patients with normal and normalized CRP could be indicative of a better CPI efficacy.

## Conclusions

Overall, patients with normal and normalized CRP had a favorable prognosis compared to patients with non-normalized CRP. Therefore, not only patients with low baseline CRP levels might benefit from CPI-based 1L therapy, but also patients with initially elevated CRP levels, if their CRP level decreases during the early treatment phase. As a standard laboratory parameter, CRP kinetics could be used to predict CPI efficacy as it can be easily implemented into clinical routine to facilitate therapy monitoring.

### Supplementary Information

Below is the link to the electronic supplementary material.

Supplementary file1 (PDF 53 KB)

## Data Availability

The datasets generated during and/or analyzed during the current study are available from the corresponding author on reasonable request.
