# Supplementary material for: Early kinetics of C-reactive protein as prognosticator for survival in a real-world cohort of patients with metastatic renal cell cancer under first-line therapy with immune checkpoint inhibitors
Source: Clin Transl Oncol. 2023 Sep 11;26(5):1117–28. doi: 10.1007/s12094-023-03317-z (PMC11026221; doi:10.1007/s12094-023-03317-z)
Supplement: Supplementary file 1 — Supplementary file1 (PDF 53 KB) [file 12094_2023_3317_MOESM1_ESM.pdf]

Early kinetics of C-reactive protein as prognosticator for survival in a real-world cohort of patients with metastatic renal cell cancer under first-line therapy with immune checkpoint inhibitors

Vayda Schüttke (1)‡, Cathrin Kusiek (1)‡, Susanne Fuessel (1,2), Christian Thomas (1), Bjoern Thorben Buerk (1)¶, Kati Erdmann (1,2)¶*

(1) Technische Universität Dresden, Faculty of Medicine and University Hospital Carl Gustav Carus, Department of Urology, Dresden, Germany

(2) German Cancer Consortium (DKTK), Partner Site Dresden, Dresden and German Cancer Research Center (DKFZ), Heidelberg, Germany

‡ Equal contributors

¶ Joint senior authors

* Corresponding author: kati.erdmann@ukdd.de

Table S1 CRP specifics according to the time point of 1L start

Parameter	Category	Total cohort	Time point of 1L start				P value [b]
			1st year	2nd year	3rd year	4th year	
Period of 1L start		03/2019-10/2022	03-12/2019	01-12/2020	01-10/2021	01-10/2022	
Patients (n)		61 (100.0%)	11 (18.0%)	16 (26.2%)	14 (23.0%)	20 (32.8%)	
Baseline CRP (mg/l)	Median (range)	20.2 (0.7-241.9)	25.10 (2.3-241.9)	16.10 (1.9-110.2)	8.40 (0.7-197.2)	19.40 (1.0-127.6)	0.365
Nadir CRP (mg/l) [a]	Median (range)	4.8 (0.6-133.9)	16.80 (1.0-133.9)	6.60 (1.3-32.7)	2.65 (1.3-28.8)	5.20 (0.6-62.4)	0.202
CRP measurements (n) [a]	Median (range)	7 (2-11)	7 (2-11)	7 (5-11)	6 (5-11)	6 (3-11)	0.192

[a] Within first three months after initiation of 1L therapy. [b] Comparison between years of 1L start by Kruskal-Wallis test.

Table S2 Baseline data on kidney and liver function in the total cohort and sub-cohorts according to early CRP kinetics

**Parameter**	**Category**	**Total cohort**	**Early CRP kinetics**			**P value** [b]
			**Normal**	**Normalized**	**Non-Normalized**	
Patients (n)		61 (100.0%)	23 (37.7%)	25 (41.0%)	13 (21.3%)	
Creatinine (µmol/l) [a]	Median (range)	96.5 (49-163)	111.0 (58-163)	93.0 (68-139)	81.0 (49-154)	**0.023**
eGFR (ml/min/1.73m^2) [a]	Median (range)	64.0 (29-90)	56.0 (36-90)	66.0 (46-90)	79.5 (29-90)	**0.055**
ALAT (µmol/s*l) [a]	Median (range)	0.32 (0.12-1.48)	0.39 (0.18-1.48)	0.29 (0.12-1.42)	0.30 (0.13-1.23)	0.132
ASAT (µmol/s*l)	Median (range)	0.37 (0.15-2.72)	0.40 (0.23-1.61)	0.32 (0.15-1.01)	0.37 (0.16-2.72)	0.103
GGT (µmol/s*l)	Median (range)	0.60 (0.21-9.46)	0.51 (0.21-9.46)	0.61 (0.35-8.14)	1.08 (0.23-4.16)	0.197
Bilirubin (µmol/l) [a]	Median (range)	5.45 (2.5-13.7)	5.90 (2.5-11.7)	5.90 (2.7-13.7)	4.90 (2.5-10.1)	0.349

[a] Data were only available for 60 instead of 61 patients at start of 1L therapy. [b] Comparison between groups of early CRP kinetics by Kruskal-Wallis test. Significant p values (<0.05) and statistical trends (p ≥0.05 & <0.1) are displayed in bold. Abbreviations: ALAT - alanine transaminase, ASAT - aspartate transaminase, eGFR - estimated glomerular filtration rate, GGT - gamma-glutamyltransferase

a

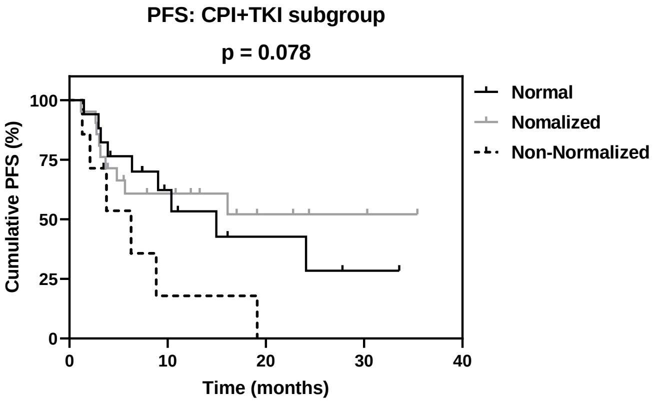

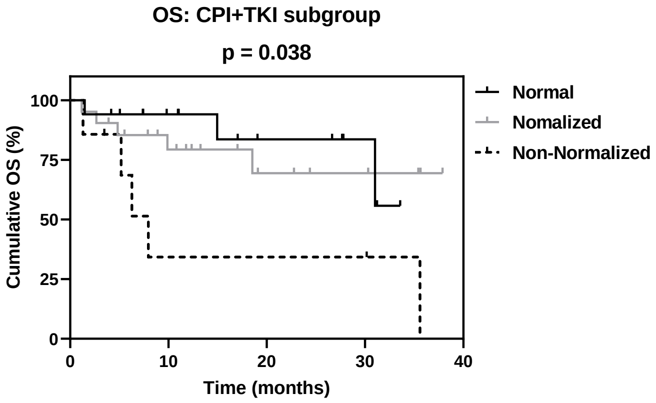

Early CRP kinetics	n (events)	1-year PFS	2-year PFS	Median PFS
Normal	17 (9)	53%	41%	15.0 mo.
Normalized	21 (9)	60%	52%	n.r.
Non-Normalized	7 (6)	18%	0%	6.3 mo.

Early CRP kinetics	n (events)	1-year OS	2-year OS	Median OS
Normal	17 (3)	94%	83%	n.r.
Normalized	21 (5)	79%	68%	n.r.
Non-Normalized	7 (5)	35%	35%	8.0 mo.

b

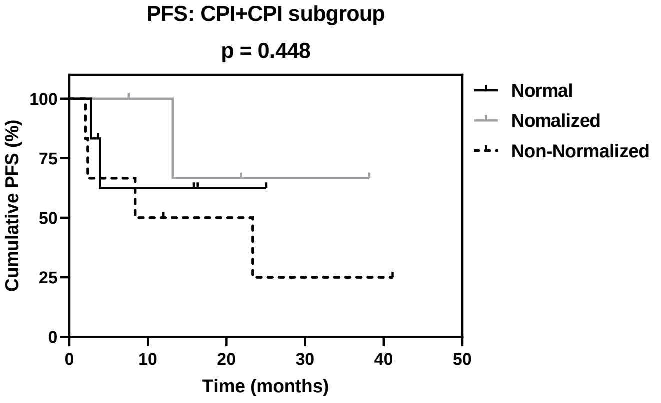

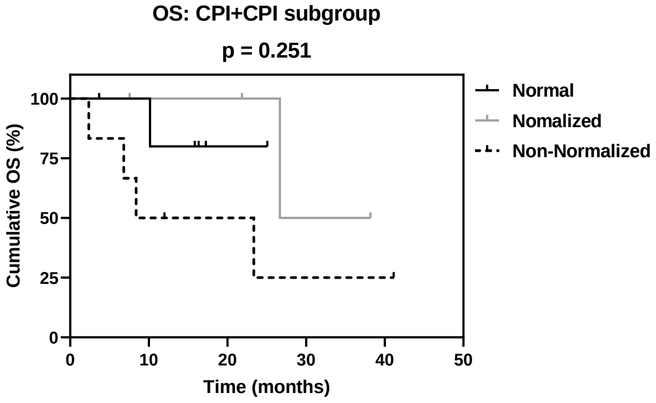

Early CRP kinetics	n (events)	1-year PFS	2-year PFS	Median PFS
Normal	6 (2)	64%	64%	n.r.
Normalized	4 (1)	100%	67%	n.r.
Non-Normalized	6 (4)	48%	24%	8.4 mo.

Early CRP kinetics	n (events)	1-year OS	2-year OS	Median OS
Normal	6 (1)	80%	80%	n.r.
Normalized	4 (1)	100%	100%	26.7 mo.
Non-Normalized	6 (4)	46%	23%	8.4 mo.

Fig. S1 Association of early CRP kinetics in the treatment subgroups of **(a)** CPI+TKI and **(b)** CPI+CPI with PFS and OS after initiation of CPI-based 1L therapy of mRCC patients. The table beneath each Kaplan-Meier curve includes the number of patients and events in each category as well as the respective median survival times, 1- and 2-year survival rates. P values were calculated by the log-rank test. Abbreviations: mo.: months; n.r.: not reached

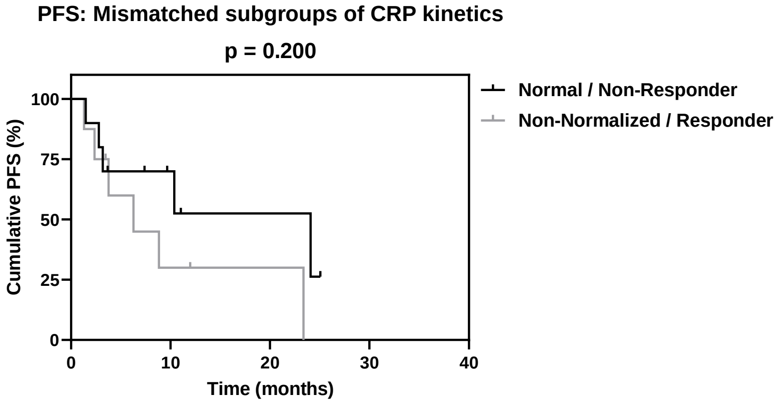

Mismatched subgroups of CRP kinetics	n (events)	1-year PFS	2-year PFS	Median PFS
Normal / Non-Responder	10 (5)	53%	53%	24.1 mo.
Non-Normalized / Responder	8 (6)	26%	0%	6.3 mo.

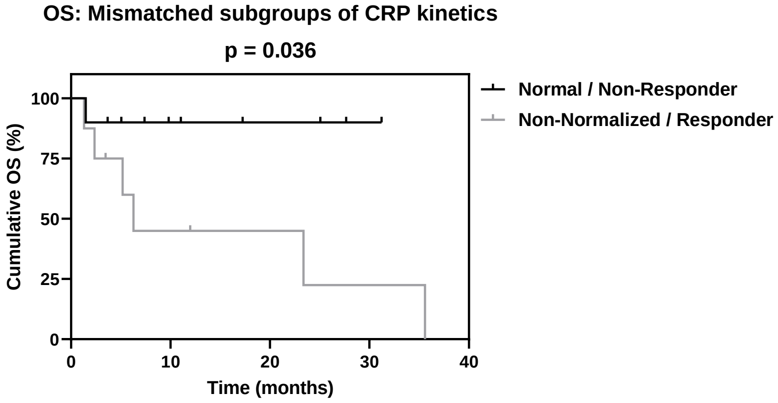

Mismatched subgroups of CRP kinetics	n (events)	1-year OS	2-year OS	Median OS
Normal / Non-Responder	10 (1)	89%	89%	n.r.
Non-Normalized / Responder	8 (6)	43%	21%	6.3 mo.

Fig. S2 Association of potentially mismatched subgroups of CRP kinetics (**Table 5**) with PFS and OS after initiation of CPI-based 1L therapy of mRCC patients. The table beneath each Kaplan-Meier curve includes the number of patients and events in each category as well as the respective median survival times, 1- and 2-year survival rates. P values were calculated by the log-rank test. Abbreviations: mo.: months; n.r.: not reached
